# Peculiarities in coronary sinus anatomy: implications for successful cannulation from an autoptic study

**DOI:** 10.1093/europace/euab108

**Published:** 2021-04-17

**Authors:** Sylwia Sławek-Szmyt, Krzysztof Szmyt, Czesław Żaba, Marek Grygier, Maciej Lesiak, Aleksander Araszkiewicz

**Affiliations:** 1 1st Department of Cardiology, Poznan University of Medical Sciences, Dluga Street ½, 61-848 Poznan, Poland; 2 Department of General, Endocrine and Gastrointestinal Oncology Surgery, Poznan University of Medical Sciences, Przybyszewski Street 49, 60-355 Poznan, Poland; 3 Department of Forensic Medicine, Poznan University of Medical Sciences, Swiecicki Street 6, 60-789 Poznan, Poland

**Keywords:** Coronary sinus, Thebesian valve, Vieussens valve, Ostium, Cannulation, Vein of Marshall, Tributaries

## Abstract

**Aims:**

The number of cardiovascular procedures using the coronary sinus (CS) as a gateway is constantly increasing. The present study aimed to define specific structures within CS, which could potentially complicate CS cannulation and to develop a new Thebesian valve (TV) classification system.

**Methods and results:**

The study was performed on 560 consecutive unfixed cadaveric hearts during routine autopsy examination (1–3 days post-mortem). Basic CS dimensions were measured and the presence and dimensions of the TV and the Vieussens valve (VV) were assessed. Thebesian valves were classified according to their morphology into six main types: remnant fold, semilunar, fenestrated, chord, fused strands, and mixed shaped. The median age of hearts was 48 years (range 16–95 years), and 38.9% were female. Thebesian valve was present in 79.5%. The most common TV type was semilunar (54%) followed by fenestrated (8.2%), remnant fold (5.5%), fused strands (4.8%), chord (4.0%), and mixed shaped (3.0%). In 1.1% of hearts, TV totally covered the coronary sinus ostium (CSO). The VV was detected in 67.9%. Potentially occlusive VV was found in 1.1% hearts and in all of which it coexisted with obstructive TV. The median CSO area was 87.9 mm^2^ [interquartile range (IQR): 56.5–127.1 mm^2^] and median CS length was 38 mm (IQR: 29.5–45 mm). The CSO area and CS length correlated with each other and with the right atrium’s dimensions.

**Conclusion:**

We identified six types of TVs, among which only 1.1% TVs caused total occlusion of CSO. The obstructive TV co-existed with potentially occlusive VV what might hinder CS cannulation.

**What’s new?**

- This is the largest study to be performed on unfixed heart specimens to assess the complete coronary sinus (CS) anatomy.
- The study proposes a new classification of the Thebesian valve (TV), which includes six morphological types.
- An occlusive TV coexisted with an obstructive Vieussens valve in 1.1% of the examined hearts.
- In hearts with no TV, the CS ostium area and the CS length correlated significantly with each other and with the right atrium’s transverse and longitudinal diameters.

## Introduction

The coronary sinus (CS) is the central vein of the coronary venous system, which runs in the posterior part of the coronary groove as a continuation of the great cardiac vein from the Vieussens valve (VV) to its ostium located in the inferomedial part of the right atrium, between the inferior vena cava orifice and the inferior tricuspid annulus.^1^,^2^

The CS opening is frequently covered by a fold of endocardial tissue termed the Thebesian valve (TV). The role of the TV in normal physiology is not fully understood, but it is believed that it may prevent blood regurgitation into the CS during the contraction of the atrium.^1^ The TV is characterized by a wide diversity of shapes. Previous studies have proposed incomplete and ambiguous TV classifications based on small samples.^2^^,^^3^

The CS has increasingly been used as a passage for various cardiovascular procedures: left ventricular lead placement in cardiac resynchronization therapy, mapping and ablation of different arrhythmias, percutaneous mitral annuloplasty, retrograde cardioplegia delivery, coronary venous retroperfusion, and targeted drug delivery or placement of a coronary sinus reducer in refractory angina.^4^ However, CS cannulation may fail due to anatomical variations, anomalies, malformations, and irregularities. A displaced CS catheter may cause life-threatening complications, such as cardiac tamponade, myocardial damage, and right ventricle haematoma.^5^

Despite the high clinical importance of the coronary venous vasculature, knowledge of its anatomy remains limited. Cadaveric studies on formalin-fixed hearts are still the most reliable source of anatomical knowledge of the venous system of the heart. Additional data have been obtained by imaging-based studies using venography, cadaveric coronary vein endoscopy, cardiac computed tomography, or cardiac magnetic resonance.^6–8^

The purpose of this study was to assess the CS anatomy with regard to the coronary sinus ostium (CSO). In particular, we aimed to identify specific structures within the CS that could complicate electrophysiological and cardiac invasive procedures by examining human cadaveric hearts that had not undergone formalin fixation. We also intended to develop a new TV classification system.

## Methods

This cross-sectional observational study was performed between June 2014 and December 2019 at the Department of Forensic Medicine of Poznan University of Medical Sciences, Poznan, Poland. The study was performed on 560 consecutive cadaveric hearts during routine autopsies. All hearts were examined 1–3 days post-mortem, and none had been previously preserved with formalin. The exclusion criteria were as follows: cardiac cause of death, cardiac injury, any anatomical heart anomalies, a medical history of severe cardiac pathologies (such as myocardial infarction with reduced ejection fraction or storage diseases), previous cardiac surgery or invasive intracardial procedures, and macroscopic signs of putrefaction. Demographic and clinical data were obtained from the departmental records and available medical histories. The study was approved by the Ethical Committee of Poznan University of Medical Sciences (approval number 529/2014).

All examined hearts were dissected from bodies in a routine manner along with parts of the great vessels. First, macroscopic measurements of basic heart parameters, including heart length and width, right atrium (RA) longitudinal and transverse dimensions, and left ventricle (LV) and right ventricle (RV) thickness, were performed. The heart’s length was measured from the apex to the superior border of the base in the middle. The width was measured at the widest part of the transverse diameter. The RA long axis was measured from the midpoint of the medial aspect of the tricuspid annulus to the superior point of the atrial wall in the plane of the interatrial septum. The transverse dimension of the RA was measured as the greatest distance from the septal to the free wall surfaces of the RA in a plane perpendicular to the long axis. The thickness of the free walls of the LV and RV and the interventricular septum was measured at the mid-ventricular level. All measurements were performed with an electronic calliper (Topex, 31C624; accuracy: 0.02 mm).

### Coronary sinus anatomy assessment

**Figure 1:**
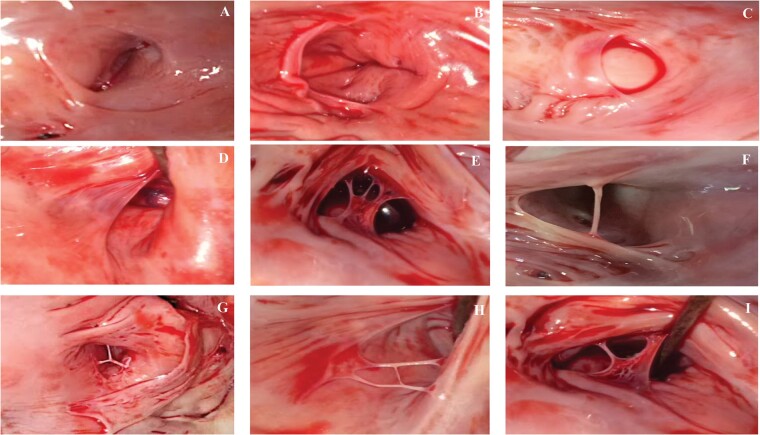
Classification of the TV types. (*A*) Absence of the TV (–), (*B*) remnant fold (type I), (*C*) semilunar nonobstructive (type IIa), (*D*) semilunar possible obstructive (type IIb), (*E*) fenestrated (type III), (*F*) chord (type IV), (*G*) fused strands Y-shaped (type Va), (*H*) fused strands H-shaped (type Vb), and (*I*) mixed shaped (type VI). TV, Thebesian valve.

To examine the right atrium anatomy in detail, a long lateral section from the apex of the RV to the orifice of the superior vena cava was made, cutting the posterior cuspid of the tricuspid valve. The morphology of the CSO and the presence and morphology of the TV were recorded in all heart specimens. The vertical and transverse diameters of the CSO were also measured. In all examined hearts, the CSO had an elliptic shape. For this reason, the CSO area was calculated according to the commonly used formula for the surface area of an ellipse (π × half the vertical dimension × half the transverse dimension). The craniocaudal and transverse dimensions of the TV were also measured. The TV was studied with particular attention to its morphology and location and its coverage percentage of the CSO. The coverage percentage was calculated according to the formula of Mehra: maximum transverse dimension of the TV/transverse diameter of the CSO × 100.^5^ A potentially obstructive TV, which may interfere with the cannulation of the CS, was defined as a non-fenestrated valve covering >75% of the CSO area. Based on our own data, we developed a new TV classification according to its morphology. We identified six major types of the TVs: remnant fold (I), semilunar (II), fenestrated (III), chord (IV), fused strands (V), and mixed shaped (VI). Details are shown in *Table 1* and *Figure 1*.

**Table 1:** Classification of the TV with the detailed frequency

Type	Name	Description	Frequency	
			*n* = 560	100%
–	None	Absence of the TV	115	20.5
I	Remnant fold	Residual endocardial flap around the perimeter, that covers <10% of the total CSO area	31	5.5
II	Semilunar	Crescentic endocardial flap covering the CSO in variable degree	302	54.0
IIa	Nonobstructive	The valve covers <75% of the CSO	291	52.0
IIb	Possible obstructive	The valve covers >75% the of CSO	11^a^	2.0^a^
III	Fenestrated	Cribriform, net-like valve mostly semilunar shaped	46	8.2
IV	Chord	A simple endocardial band mostly in middle of the CSO	22	4.0
V	Fused strands	Connected in different manner endocardial strands localized mostly midline	27	4.8
Va	Y-shaped strands	Endocardial strands fused into Y-letter	11	2.0
Vb	H-shaped strands	Endocardial strands fused into H-letter	16	2.8
VI	Mixed shaped	Co-existence of two different TV types from 2 to 5	17	3.0

TV, Thebesian valve; CSO, coronary sinus ostium.

a Among subtype IIb six TVs (1.1% of heart specimens) covered 100% of the CSO.

The CS was opened by a longitudinal incision along its free wall without touching the TV if present. The presence and morphology of the VV were also noted including the number of cusps and their shape (concave, flat, or diminutive). If a VV was detected, the diameter of each leaflet was measured, and the coverage percentage of the CS was calculated according to the above-mentioned Mehra formula.^5^ Like a potentially obstructive TV, a potentially occlusive VV was defined as a valve covering at least 75% of the CS lumen. The ostium of the vein of Marshall (VOM) was also identified, and its relation to the VV was evaluated. The length of the CS was measured as the distance between the VV and the CSO. In cases where the VV was absent, the length of the CS was measured from the ostium of the VOM marked as its starting point. The diameter of the CS was measured at three points: at the beginning, at the point of entry of the middle cardiac vein, and at the ostium. All linear measurements were performed with the heart held in the anatomical position using the above-mentioned electronic calliper twice by two researchers independently (S.S.-S. and K.S.) to eliminate significant errors. The mean value of each measurement was calculated for subsequent analyses. To simulate CS cannulation in potentially obstructive TVs, 3-, 5-, and 7-French electrophysiology diagnostic catheters (Boston Scientific, Marlborough, MA, USA) were used.

**Figure 2:**
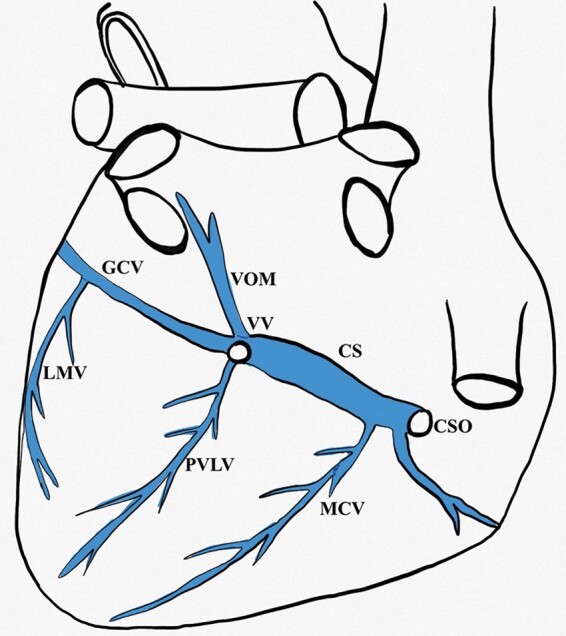
The schematic anatomy of coronary venous system. CS, coronary sinus; CSO, coronary sinus ostium; GCV, great cardiac vein; LMV, lateral marginal vein; MCV, middle cardiac vein; PVLV, posterior vein of left ventricle; VOM, vein of Marshall; VV, Vieussens valve.

**Figure 3:**
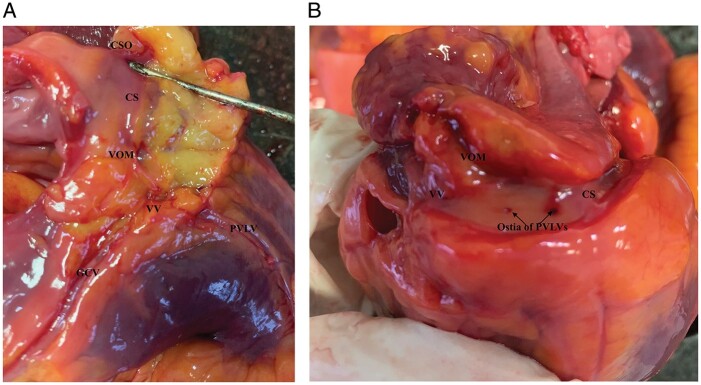
Photographic presentation of the CS anatomy. (*A*) The confluence of the VOM and the GCV with the presence of concave tricuspid VV and (*B*) the ostia of PVLVs draining into CS. CS, coronary sinus; CSO, coronary sinus ostium; GCV, great cardiac vein; PVLV, posterior vein of left ventricle; VOM, vein of Marshall; VV, Vieussens valve.

The presence and number of veins draining into the CS (CS tributaries) were also evaluated. The detailed anatomy of CS tributaries is shown in *Figures 2 and 3*. Moreover, for each CS tributary from the LV territory, the following measurements were performed: ostium diameter measured at the point of CS entry and length, defined as the distance between the ostium of the coronary vein and the first dichotomous division of the vessel measured along the course of the vein.

### Statistical analysis

Statistical analysis was performed using Statistica version 13.7. The appropriate study sample size was estimated using a proportion based on the largest previous study.^9^ The sample proportion level was set to 0.63 with a 0.95 confidence level, an alpha level of 0.05, and a marginal error of 0.04. Continuous data were expressed as medians and interquartile ranges (IQRs). Categorical data were expressed as numbers and percentages. The normality of data distribution was assessed with the Shapiro–Wilk test. Categorical variables were compared using the two-tailed Fisher’s exact test or the *χ*^2^ test as appropriate, and continuous variables were analysed using the nonparametric Mann–Whitney *U* test (no variables were normally distributed). Interactions between the variables were evaluated using Spearman’s correlation test. A two-tailed alpha of 0.05 and a *P*-value of <0.05 were considered statistically significant.

## Results

Of the 560 hearts, 38.9% were from female cadavers. The median age of the autopsied subjects was 48 years (range: 16–95 years), and the median body mass index (BMI) was 23.6 kg/m^2^ (IQR: 20–27 kg/m^2^). The median weight of the hearts was 370 g (IQR: 314–401 g). There were statistically significant differences in most macroscopic parameters between female and male cadavers. Details are presented in *Table 2*.

**Table 2:** Basic quantitative parameters of female and male hearts

Characteristic	All	*n* = 560	Female, *n* = 218		Male, *n* = 342		*P-*value
	Median	IQR	Median	IQR	Median	IQR	
Age (years)	48	33.5–65.5	55	37–80	41.5	33–60	<0.0001
Body mass index (kg/m^2^)	23.6	20–27	21.7	18.9–25.5	24.6	22.2–27	<0.0001
Body surface area (m^2^)	1.8	1.6–1.93	1.6	1.5–1.6	1.9	1.8–2.1	<0.0001
Heart weight (g)	370	314–401	318	251–375	382	326–452	<0.0001
Heart length (mm)	105	95–110	100	95–105	105	100–120	<0.0001
Heart width (mm)	100	90–110	95	80–100	100	90–115	<0.0001
RA longitudinal diameter (mm)	4.1	3.9–4.3	4	3.7–4.2	4.2	4–4.4	<0.0001
RA transverse diameter (mm)	3.6	3.4–3.8	3.5	3.2–3.7	3.7	3.5–3.9	<0.0001
LV thickness (mm)	11	10–12	10	9–11	11	10–12	<0.0001
RV thickness (mm)	3	3–4	3	3–4	3	3–4	0.61

IQR, interquartile range; LV, left ventricle; RA, right atrium; RV, right ventricle.

The TV was present in 79.5% (*n = *445) of the hearts. In terms of shape, the most common was type II—semilunar (54%), followed by type III—fenestrated (8.2%), type I—remnant fold (5.5%), type V—fused strands (4.8%), type IV—chord (4%), and type VI—mixed shaped (3%) (*Table 1*). The median percentage of CS coverage by the TV (valve types IIa, IIb, III, and VI) was 55% (IQR: 40–73%). The potentially obstructive TV (type IIb) was identified in 2% (*n = *11) of all specimens including 1.1% (*n* = 6) that covered 100% of the CSO. Using the smallest (3-F) electrophysiology catheter, we were able to cannulate all fenestrated and mixed-shaped valves, as well as two of the six TVs completely covering the CSO.

The median length of the CS in the entire sample was 38 mm (IQR: 29.5–45 mm) and was significantly greater in male specimens (44 vs. 31 mm; *P *<* *0.0001). There were also significant differences between male and female hearts in median maximal diameter of the CS (24 vs. 22 mm; *P *<* *0.0001) and median CSO area (102 vs. 60.5 mm^2^; *P *<* *0.0001). *Table 3* presents the detailed results of macroscopic measurements of the heart structures.

**Table 3:** Coronary sinus and coronary veins characteristics comparison between female and male hearts

Characteristic	All	*n* = 560	Female, *n* = 218		Male, *n* = 342		*P-*value
	Median	IQR	Median	IQR	Median	IQR	
CS maximal diameter (mm)	24	19–30	22	18–29	24	20–30	0.001
CS length (mm)	38	29.5–45	31	28–37	44	35–49	<0.0001
CSO horizontal dimension (mm)	12	8–15	10	7–15	12	8–17	<0.0001
CSO vertical dimension (mm)	10	8–12	9	7–10	11	9–13	<0.0001
CSO area (mm^2^)	87.9	56.5–127.1	60.5	42.4–98.1	102	75.4–146.8	<0.0001
Indexed CSO area (mm^2^/m^2^)	150.9	99.8–244.1	99.8	69.2–157.1	203.4	139.3–282.1	<0.0001
PVLV1 ostium diameter (mm)	0.9	0.8–1.1	0.8	0.8 –1.1	0.9	0.9–1	0.74
PVLV1 length (mm)	39	33–44	39	34–40	42	33–48	0.003
PVLV2 ostium diameter (mm)	1	0.9–1.1	1	0.9–1.1	1	1–1.1	0.09
PVLV2 length (mm)	41.5	35.5–56.5	41.5	36.5–42.5	42.5	35.5–50.5	0.003
LMV ostium diameter (mm)	1.1	1–1.2	1.1	1–1.2	1.1	1–1.2	0.15
LMV length (mm)	43	37–48	43	38–47	44	37–52	0.016

CS, coronary sinus; CSO, coronary sinus ostium; IQR, interquartile range; LMV, lateral marginal vein; PVLV1, first posterior vein of left ventricle (from CSO); PVLV2, second posterior vein of left ventricle (from CSO).

The CS length positively correlated with the RA transverse diameter (*r *=* *0.37; *P *<* *0.0001), RA longitudinal diameter (*r *=* *0.34; *P *<* *0.0001), and CSO area (*r *=* *0.38; *P *<* *0.0001). Similarly, there were strong correlations between the CSO area and the RA transverse (*r *=* *0.75; *P *<* *0.0001) and longitudinal (*r *=* *0.74; *P *<* *0.0001) diameters. The detailed correlation test results are displayed in *Table 4*.

**Table 4:** Correlations between CS parameters and basic heart parameters

Characteristics	CSO area		CS length		CS maximal diameter		Number of CS tributaries	
	*r*	*P*-value	*r*	*P*-value	*r*	*P*-value	*r*	*P*-value
Age	−0.07	0.062	−0.07	0.064	0.27	<0.0001	0.17	0.06
Body mass index	0.04	0.38	0.25	<0.0001	0.1	0.02	−0.07	0.07
Body surface area	0.27	<0.0001	**0.43** ^a^	**<0.0001**	0.03	0.38	−0.06	0.15
Heart weight	0.14	0.0008	0.23	<0.0001	0.29	<0.0001	−0.1	0.09
Heart length	0.17	<0.0001	0.2	<0.0001	0.23	<0.0001	0.7	0.1
Heart width	−0.07	0.08	0.06	0.13	0.22	<0.0001	−0.02	0.62
RA longitudinal diameter	**0.74** ^b^	**<0.0001**	**0.34** ^a^	**<0.0001**	0.05	0.29	−0.07	0.09
RA transverse diameter	**0.75** ^b^	**<0.0001**	**0.37** ^a^	**<0.0001**	0.04	0.28	−0.07	0.1
LV thickness	0.1	0.01	0.16	0.0001	0.26	<0.0001	−0.05	0.26
RV thickness	−0.22	<0.0001	−0.01	0.85	0.25	<0.0001	0.12	0.4
CS length	**0.39** ^a^	**<0.0001**	—	—	−0.05	0.26	0.07	0.064

CS, coronary sinus; CSO, coronary sinus ostium; RA, right atrium.

Significant correlations are set in bold.

a Significant positive correlation.

b Strong positive correlation.

The CS most frequently had four tributaries from the LV territory (*n *=* *207; 37%), followed by five (*n *=* *157; 28%), three (*n *=* *146; 26%), six (*n *=* *28; 5%), and two tributaries (*n *=* *22; 3.9%). There were no significant differences in the number of tributaries from the LV territory in terms of sex, age, BMI, or basic heart parameters (*Table 4*).

The VV was present in 67.9% of the heart specimens. Most frequently, it was composed of two concave cusps (32.1%). Details are shown in *Table 5*. The median percentage of CS coverage by the VV was 41.2% (IQR: 35–49%). A potentially occlusive VV was found in six (1.1%) hearts, in all of which it coexisted with a TV completely covering the CSO. Using a 3-F electrophysiology catheter, we were able to catheterize four of the six potentially occlusive VVs.

**Table 5:** Frequency of each type of the VV

VV morphology	*n* = 560	100%
None	180	32.1
Total	380	67.9
Unicuspid	104	18.6
Concave	64	11.5
Flat	40	7.1
Bicuspid	236	42.1
Concave	180	32.1
Flat	56	10
Tricuspid	10	1.8
Concave	10^a^	1.8^a^
Flat	—	—
Diminutive	30	5.4

a Among subtype tricuspid concave six VVs (1.1% of heart specimens) cover >75% of the CS lumen.

VV, Vieussens valve.

## Discussion

Cardiovascular procedures using the CS as a gateway have significantly increased over the past decades, and successful CS cannulation constitutes a significant challenge in modern cardiology. Data suggest that up to 10% of all CS catheterizations fail.^10^

One of the important factors determining the success of CS cannulation is the CSO area. To our knowledge, this is the first study to perform measurements on unfixed heart specimens, close to *in vivo* conditions, thus avoiding the tissue shrinkage that occurs after fixation. In hearts where the TV was absent, the median CSO area was 97.9 mm^2^. The CSO area strongly correlated with the CS length. According to the authors it could be related to the increased blood flow causing also stretching of myocardial sleeve covering the CS and thus increasing the CS length and the CSO area. This finding may be partially explained by sex differences. Most heart parameters were significantly higher in male than in female hearts. Interestingly, the CSO area and CS length positively correlated with the RA transverse and longitudinal dimensions. In living hearts, the CS parameters and RA dimensions change with the heartbeat, with minimum values during the atrial systole. A previous study assessing patients with primary pulmonary hypertension using echocardiography revealed a strong correlation between RA size and CS diameter. However, the CS diameter was not related to the pulmonary arterial or RV pressure or to the extent of tricuspid regurgitation, suggesting that the CS might behave as an extension of the RA, like the inferior vena cava.^11^ Our results are in line with these findings. Awareness of the correlations between structures of the CS may facilitate the pre-procedural planning and selection of appropriate devices and reduce the procedure time and the risk of potential complications.

The CSO is frequently covered by the TV, which exhibits high morphological variability. Studies with small sample sizes have estimated a prevalence of TV ranging from 62% to 88%.^12^^,^^13^ In our study, the TV was present in 79.5% of the specimens, which is in line with previous findings. On the other hand, Anh *et al*.,^14^ using *in vivo* visualization of the TV with an illuminated endocardial fibreoptic endoscope, reported that the TV was present in 54% of the examined hearts. This lower prevalence may have resulted from CSO dilatation caused by the blood flow during the cardiac cycle and the difficulty in visualizing residual or thin TVs.

In this study, we observed various morphologies of the TV. Although attempts to describe TV variability have previously been made,^2^^,^^3^^,^^15^ we found that existing descriptions are insufficient. For this reason, we decided to create our own classification. We distinguished six main TV types and provided descriptions and images of each. It should be emphasized that our observations were based on the examination of the biggest heart specimens in an unfixed state during autopsies. The most frequent type was semilunar nonobstructive TV (type IIa), detected in 52% of the specimens. This is consistent with previous studies also reporting the semilunar valve as the most common type.^2^^,^^3^^,^^14–16^ The main types newly described in this study are fused strands and mixed shaped. We discerned these two additional types due to their unique shape and structure.

Several studies have shown that the TV may obstruct the entrance to the CS and prevent its cannulation.^2^^,^^10^^,^^15^^,^^16^ A potentially obstructive TV is identified mainly based on the percentage of CSO coverage. Coverage of at least 75% has been considered the cut-off.^10^^,^^15^^,^^16^ In this study, using the same criterion, we identified 2% of TVs as potentially obstructive (type IIb); however, only in 1.1% of all specimens TV type IIb completely covered the CSO. In previous studies, the prevalence of an obstructive TV ranged from 16% to 36%.^10^^,^^15^ However, Hołda *et al*.^2^ suggested that only TVs completely covering the CSO or protruding beyond the CSO contour may render CS cannulation intractable or impossible. Ghosh *et al*.^15^ defined an obstructive TV not only according to the percentage of CSO coverage (≥75%) but also according to the composition of the TV: no fenestrated muscular, fibrous, or fibromuscular. However, research has shown that most valves with a fibrous or fibromuscular constitution are from heart specimens with evidence of organic disease. This suggests that some TV types may become obstructive in the course of an underlying organic heart disease. Interestingly, Hill *et al*.^7^ demonstrated that in some cases of fenestrated TVs, the fenestration was large enough for the passage of a catheter, but the catheter caused an inward deflection of the TV, resulting in unsuccessful CS catheterization. The authors also showed that not only some TV types may obstruct or complicate the cannulation of the CSO but also the movement of the TV during the cardiac cycle towards the RA may complicate CS catheterization.^7^ In this study, we were able to catheterize all fenestrated and mixed-shaped TVs. Moreover, we managed to cannulate two of the six TVs completely covering the CSO.

Besides an obstructive TV, other obstacles may prevent successful CS cannulation. A previous study reported that the presence of a prominent VV may be one of the main obstacles to advancing the catheter into a great cardiac vein.^17^ The prevalence of the VV in cadaveric studies ranges widely (8–78%).^6^^,^^9^ In this study, the VV was present in 67.9% of the specimens. Most commonly, the VV was bicuspid concave (32.1%). Studies on the detailed morphology of the VV are sparse. Zawadzki *et al*.^6^ found double leaflet, concave VVs in 16 of 50 (32%) examined cadaveric hearts. Żabówka *et al*.^8^ reported that the most common type was single leaflet, concave (46.7%). The VVs observed in our study were mostly small and did not obstruct the lumen of the CS; only in 1.1% of the hearts did they cover >75% of the CS lumen. Similarly, Żabówka *et al*.^8^ reported the presence of an occlusive VV in 1.4% of the examined hearts.

It seems reasonable to identify any obstacles preoperatively and prepare accordingly for the coronary venous advancement in case of strong suspicion of complications during CS cannulation. Recently, a computed tomography study was conducted to address this need and to examine whether imaging modalities could be considered in cases of failure of conventional VV intubation. However, the detection rate of VV by computed tomography was significantly lower than that achieved with cadaveric findings. In computed tomography imaging, the VV covered more of the CS lumen in comparison to cadaveric measurements (VV/CS ratios of 0.24 and 0.72, respectively).^8^

It should be emphasized that the presence of an obstacle does not rule out the possibility of successful CS catheterization. CS venous angioplasty and stenting have been proposed as promising strategies. Oto *et al*.^18^ reported that 6.2% of patients required coronary venoplasty for the placement of the left ventricular lead, and 1.2% required coronary stenting to facilitate the lead placement. Kumagai *et al*.^19^ demonstrated the value of the anchor balloon technique for left ventricular lead insertion in patients with angulated and tortuous coronary sinus branches.

The CS may be easily injured during interventional procedures. The reported incidence of coronary sinus rupture or dissection is about 1%.^20^ CS injuries are difficult to repair and can be fatal. One of the possible causes of damage is the use of excessive force while advancing a catheter through a CS with an occlusive TV or VV. Detailed knowledge of the diversity of the CS anatomy will help anticipate impediments during invasive procedures. The use of imaging modalities, especially direct endoscopic visualization, in cases of difficult CS cannulation may help adjust the implanting strategy, for example, by changing the approach direction or using a softer-tipped sheath.^14^

### Limitations

This study has several limitations. Firstly, it was conducted during autopsies, that is, in the absence of physiologic *in vivo* haemodynamic conditions, which may have caused measurement bias. The dimensions and shape of coronary venous structures may change during the cardiac cycle. In haemodynamic conditions, the behaviour of the TV and VV may cause difficulties that we may have been unable to accurately depict. Second, we studied heart specimens obtained from patients without cardiovascular disease, who are not representative of the patients typically requiring CS cannulation. Therefore, we were unable to assess coronary venous system discrepancies. Third, we performed only an anatomic evaluation, but microscopic correlations were not performed. However, we strongly believe that these limitations have a relatively limited impact on the overall results and conclusions of this study.

## Conclusions

This study provides unique insight into the anatomy of the CS, which might facilitate successful CS catheterization. We identified six main types of TV. Only 1.1% of the TVs in our sample caused total occlusion of the CSO. However, we found that an obstructive TV can coexist with a VV covering >75% of the CS lumen, which might hinder CS cannulation. Moreover, the CSO area and CS length positively correlated with each other and with the RA transverse and longitudinal diameters. We believe that our findings will help physicians identify patients at risk of difficult CS catheterization.

### Funding

This research did not receive any specific grant from funding agencies in the public, commercial, or not-for-profit sectors.

**Conflict of interest:** none declared.

## Data availability

The data underlying this article will be shared on reasonable request to the corresponding author.
